# Paracentesis Thoracis—Recovery

**Published:** 1871-11

**Authors:** A. N. Talley

**Affiliations:** Columbia, S. C., Professor of the Practice of Medicine in the University of South Carolina


ATLANTA

### MEDICAL AND SURGICAL JOURNAL.

NEW SERIES.

VOL. IXB ATLANTA, GEORGIA, NOVEMBER, 1871. NO. 8.

### PARACENTESIS THORACIS—RECOVERY.

BY A. N. TALLEY, M.D., COLUMBIA, S. C.,
Professor of the Practice of Medicine in the University of South Carolina.

I was consulted on the 20th of December last by Elizabeth B——, aged sixty-five years, who gave the following history of her case:

On the 10th of the previous month, she had suffered from an attack of acute pleuritis on the right side. The treatment consisted of morphia hypodermically, mercury internally, and a blister to the seat of pain. Relief followed the use of these means, and she was dismissed, convalescent, on the fifth day. Her flesh and strength did not, however, return, and her respiration grew more and more embarrassed, till finally complete orthopnœa was established.

At this stage of the case, I was desired to visit her. I found her greatly emaciated, her countenance anxious and distressed, her respiration sixty to the minute, and her pulse exceedingly small and quick, numbering one hundred and thirty-five to the minute. Inspection of the chest showed that, while the right side was distended till the intercostal depressions were no longer visible, the movements of respiration were confined to the left side. Percussion elicited universal dullness over the right side, the area of dullness extending beyond the left lateral margin of the sternum. The liver was displaced, having been forced quite out of its normal site by the depression of the diaphragm. Auscultation detected no breath-sounds or vocal vibrations on the affected side, save just over the region of the primitive bronchus, where tubal breathing and bronchophony were heard. The heart's impulse was felt, and its sounds were heard to the left of the line of the nipple.

As the suffering of the patient was extreme, and the hazard of death by apnœa imminent, I determined immediately upon the performance of thoracentesis. This was done by puncturing the chest between the seventh and eighth ribs in the direction of a vertical line drawn through the lower angle of the scapula. In lieu of the apparatus of Dr. Wyman, I employed a common Davidson's syringe, the supply tube of which I fitted to the canula after withdrawing the trochar. The result was the removal of thirty-six ounces of fluid, with an immediate relief of urgent symptoms. The respiratory murmur was presently heard over the superior portion of the lung, and the heart-sounds approximated their accustomed site. Forty-eight hours later, there was evidently a reäccumulation of the fluid, but not to such an extent as seriously to impede the vital functions. I then ordered the following:

℞—Pil. Mass. Hydrg. ℈i.

Pulv. Scillæ, Pulv. Digital, aa ℈iss.

M. ft. pil. xv.

S.—One pill three times daily, with a draught night and morning, consisting of spts. etherini nitrici, gtt. xx.; pulv. potassæ nitrt., grs. xx.; aq. camphoraæ, ℥i.

Under the use of these remedies, combined with the application of Aitken's lotion of corrossive sublimate, co. tinct. of iodine and glycerine, (vol. 2, page 670), five days sufficed to effect the absorption of the fluid, though the lung was yet imperfectly expanded, as evidenced by the limitation of the breath-sounds (mixed bronchial and vesicular) to the infra-clavicular and mammary regions. Five grains of ammonia citrate of iron and a half ounce of cod-liver oil were directed to be given three times daily, with a liberal allowance of meat, eggs and ale, instead of the beef-tea and milk which had heretofore constituted her diet.

Ten months after the operation, examination of the chest revealed contraction of the right side, with depression of the shoulder and flattening of the chest-walls on that side. Bronchial respiration and vocal fremitus were present as high as the third intercostal space; above that line, normal breathing, somewhat exaggerated, was audible. These signs, with a somewhat accelerated respiration, constituted the only remaining evidences of the former trouble.

